# Using Social Media Platforms to Raise Health Awareness and Increase Health Education in Pakistan: Structural Equation Modeling Analysis and Questionnaire Study

**DOI:** 10.2196/65745

**Published:** 2025-04-07

**Authors:** Malik Mamoon Munir, Nabil Ahmed

**Affiliations:** 1 Integral Global Consulting LLC, 3541 Habersham at Northlake Parkway Road, Building E, Tucker, GA, 30084, United States, 1 678-427-0014

**Keywords:** social media, health awareness, health education, innovation diffusion theory, structural equation modeling, disease burden, healthcare facilities, health professionals, misinformation, cost effective

## Abstract

**Background:**

Current health care education methods in Pakistan use traditional media (eg, television and radio), community health workers, and printed materials, which often fall short of reach and engagement among most of the population. The health care sector in Pakistan has not yet used social media effectively to raise awareness and provide education about diseases. Research on the impact social media can have on health care education in Pakistan may expand current efforts, engage a wider audience, and reduce the disease burden on health care facilities.

**Objective:**

This study aims to evaluate the perceptions of health care professionals and paramedic staff regarding social media use to raise awareness and educate people about diseases as a potential means of reducing the disease burden in Pakistan.

**Methods:**

The study used two-stage structural equation modeling (SEM). Data analysis used AMOS 26.0 software, adopting scales from previous literature. Four-item scales for each social media usefulness and health awareness construct and 8-item scales for health care education constructs were adopted on the basis of their higher loading in alignment with psychometric literature. A 7-point Likert scale was used to measure each item. Data collection used convenience sampling, with questionnaires distributed to more than 450 health care professionals and paramedic staff from 2 private hospitals in Lahore, Pakistan. There were 389 useful responses received. However, 340 completed questionnaires were included in the data analysis.

**Results:**

The study found that all the squared multiple correlation (SMC) values were greater than 0.30. Furthermore, convergent validity was measured using (1) standardized factor loading (found greater than 0.5), (2) average variance explained (found greater than 0.5), and (3) composite reliability (found greater than 0.7). The confirmatory factor analysis (CFA) of the measurement model indicated the fitness of the constructs (Chi-square minimum [CMIN]=357.62; CMIN/degrees of freedom [DF]=1.80; Goodness of Fit [GFI]=0.90; Adjusted Goodness of Fit Index [AGFI]=0.89; Buntler-Bonett Normed Fit Index [NFI]=0:915; Comparative Fit Index [CFI]=0:93; Root Mean Square Residual [RMR]=0:075; Root Mean Square Error of Approximation [RMSEA]=0:055). Moreover, the structural model fitness was also confirmed (CMIN=488.6; CMIN/DF=1.85; GFI=0.861; AGFI=0.893; NFI=0.987; CFI=0.945; RMR=0:079; RMSEA=0.053). Hence, the results indicated that social media usefulness has a positive and significant effect on health awareness (hypothesis 1: *β*=.669, *P*<.001), and health awareness has a positive and significant effect on health care education in Pakistan (hypothesis 2: *β*=.557, *P*<.001).

**Conclusions:**

This study concludes that health care professionals and paramedic staff in private hospitals support the use of social media to raise awareness and provide health care education. It is considered an effective tool for reducing the disease burden in Pakistan. The study results also revealed that young health care professionals are more inclined toward social media usage and express the need for legislation to support it and establish a monitoring process to avoid misinformation.

## Introduction

### Background

In Pakistan, the health care system is resource-strapped, leading to challenges in preventing infectious diseases. The country has a high maternal mortality rate (186 deaths per 100,000 live births) and a high infant mortality rate (56 deaths per 1000 live births) [1]. Many complications arise from a lack of awareness and education about prenatal care, safe delivery practices, and postnatal care [2]. Moreover, poor understanding of nutrition has led to widespread malnutrition among children and pregnant women in Pakistan [1,2]. Malnutrition in children leads to stunted growth, weakened immunity, and impaired cognitive development [2]. The prevalence of stunting in Pakistan is among the highest in the world, affecting approximately 38% of children under 5 years of age [3].

In this context, health awareness and education campaigns potentially lead to behavioral changes and alleviate the burden on health facilities [4]. Public health campaigns, particularly those focused on preventive measures like vaccination, hygiene, and lifestyle changes, have substantially reduced the incidence of various diseases [3,5]. For example, handwashing campaigns alone have reduced respiratory infections by up to 23% [6]. Awareness campaigns aimed at managing chronic conditions, such as diabetes and hypertension, through education on medication adherence and lifestyle modifications have reduced complications and hospital visits [7]. These interventions have been shown to improve health outcomes and reduce health care costs by up to 25% [8].

Among various tools and health interventions, social media has attained the most global attention to enhance health awareness and education to a wider population and discovered its significant impact in addressing health care issues [9,10]. According to social media statistics, social media users worldwide reached 5.04 billion in January 2024, representing 63% of the world’s population, with users growing 6% annually [11]. On average, social media users spend 2 hours and 23 minutes daily on social media platforms and see various content [11,12]. Social media tools, such as Facebook (Meta), TikTok (ByteDance), Twitter (subsequently rebranded as X), Instagram (Meta), and YouTube (Google), facilitate health care professionals in educating and influencing perceptions, including networking, sharing ideas, disseminating information, demonstrating, coaching, consulting, and advertising [12,13]. Existing literature in the health care sector has exhibited the transformative potential of social media and its usefulness in spreading health awareness and education [9,10,12,13].

However, the existing literature lacks comprehensive insights into the role of social media in fostering health awareness and education within developing countries, particularly Pakistan. This study addresses this gap by applying Innovation Diffusion Theory to examine the mediating effect of health awareness and empirically testing the impact of social media on health care education among health care professionals.

Therefore, the primary objective of this study is to investigate the role of social media in enhancing health awareness and its subsequent impact on health care education in Pakistan. By addressing the gap in the literature concerning the adoption of social media for health care communication in developing countries, this research aims to provide empirical evidence and practical insights for leveraging digital platforms to improve health outcomes.

This section comprehensively reviews existing literature on social media usefulness, health awareness, health care education, and health care sector challenges in Pakistan.

### Social Media Usefulness

The global adoption of social media platforms has led to scalable public health interventions, increased precision and effectiveness, and improved resource mobilization [14]. Social media has encouraged healthy behaviors and enabled informed choices through sharing tips, challenges, and success stories [9]. Health care professionals can provide accurate, evidence-based information as trusted sources [11]. Their involvement is vital due to their expertise, credibility, and ability to reach a wide audience. Campaigns through social media on vaccination, hygiene practices, mental health awareness, and other preventive measures have been helpful in countries like Germany and France [9,10].

Moreover, social media has been effectively used to combat the pandemic and other diseases during the COVID-19 pandemic [15]. The social media data were instrumental in tracking the spread of the COVID-19 virus and disseminating accurate information [16]. Campaigns on platforms such as Facebook and Twitter have been used to promote vaccination, leading to increased uptake in various regions [12]. Tailored messaging on HIV prevention for at-risk populations has shown to be more effective than generalized campaigns [13]. Moreover, existing literature has shown examples of successful public health campaigns, such as:

1. #SmearForSmear Campaign: this social media campaign aimed to raise awareness about cervical cancer screening and successfully increase screening rates by leveraging the reach of platforms such as Twitter and Instagram [17].
2. Truth Initiative: an antismoking campaign that effectively used social media to reduce teen smoking rates through engaging content and interactive platforms [18].

### Health Awareness

Health awareness is termed as “Enhancing the ability of individuals to understand and use health information to make informed decisions about their health” [13]. Efforts for health awareness are varied and multifaceted, encompassing a wide range of activities aimed at educating the public about health issues, promoting healthy behaviors, and preventing diseases [16]. Health awareness campaigns lead to early detection and timely intervention, which benefits individuals by lowering their health care expenses and reducing the financial burden on the health care system [18]. Health awareness is generally performed using mass media, social media, posters, billboards, workshops, seminars, local outreach programs, influencers, and public figures [12]. However, social media is most useful in the current era due to its cost-effectiveness and wider audience engagement [17]. Social media allows targeted messaging based on demographics, interests, and online behavior [19]. This ensures that health messages reach the people most likely to benefit from them. Moreover, the effectiveness of health awareness through social media can be measured in a few easy ways, such as the number of impressions, likes, shares, comments, hashtag usage, and so on [18,19]. Hence, medical professionals can analyze the efficacy of their awareness messages. There have been several successful health awareness campaigns conducted through social media that have achieved significant outcomes (Textbox 1).

Textbox 1. **Health care awareness campaigns.**

**ThisGirlCan (2015)**
  - Objective: Encourage women of all ages and backgrounds to be more physically active, regardless of their shape, size, or fitness level [20].
  - Execution: The campaign featured real women participating in various physical activities, promoting the message that women should feel confident about being active regardless of societal pressures or body image concerns.
  - Platforms used: Instagram, Twitter, Facebook, and YouTube.
  - Outcomes:
    - Widespread engagement: The hashtag #ThisGirlCan was used millions of times, with significant engagement from women sharing their own stories and photos.
    - Behavioral change: The campaign was credited with encouraging over 2.8 million women in the United Kingdom to become more active.
    - Long-term impact: The campaign continued beyond its initial phase, evolving into a broader movement that still influences public attitudes towards women and fitness.

**#BellLetsTalk (Ongoing)**
  - Objective: Raise awareness about mental health issues and reduce stigma around mental illness in Canada [21].
  - Execution: For every tweet using the hashtag #BellLetsTalk, every text message sent by Bell customers, and every Facebook video view, Bell Canada donates 5 cents to mental health initiatives.
  - Platforms used: Twitter, Facebook, Instagram, and Snapchat.
  - Outcomes:
    - Record participation: The 2021 campaign saw 159 million interactions, raising nearly CAD $8 million (equivalent US $5.56 million) in a single day.
    - Sustained impact: Since its inception, the campaign has raised more than CAD $121 million (equivalent US $84.5 million) for mental health initiatives.
    - Increased conversations: The campaign has significantly increased public dialogue about mental health in Canada, contributing to reducing stigma and promoting mental wellness.

### Health Care Education

Health care education refers to a deeper understanding of specific health topics, including the causes, symptoms, prevention, and treatment of diseases and how to maintain or improve health [20]. It involves having factual information and comprehension of health-related subjects [17]. Health care education encourages regular check-ups, screenings, and vaccinations, leading to early detection of diseases and more effective prevention strategies [19]. For those with chronic conditions such as diabetes, hypertension, or asthma, health care education is essential for managing symptoms, adhering to treatment plans, and avoiding complications [13].

In resource-limited economies, insufficient health care education has been reported among the population, which impedes individuals’ ability to understand, access, and apply health information effectively [21]. Moreover, low-income individuals may have less access to health resources, education, and information, which hinders their ability to acquire and apply health education [22]. Addressing these barriers in resource-limited countries requires targeted strategies such as improving health literacy, offering culturally sensitive health care education, increasing access to technology, and combating misinformation [21].

Among these targeted strategies, social media has gained the most attention in the high-income world [20]. Health care education through social media has become an increasingly effective method for reaching diverse audiences with health information. Studies suggest that health care education campaigns on social media can achieve behavioral change success rates ranging from 20% to 40% [16,17]. For instance, social media campaigns promoting COVID-19 vaccination have seen varying success, with some countries reporting a 15% to 25% increase in vaccine uptake attributable to social media efforts [15]. Moreover, some campaigns that promote health services (eg, vaccination drives or mental health counseling) report conversion rates (actual service uptake) of 5% to 20%, depending on the call to action and the ease of access to the service [18]. The existing literature has discussed a few notable health care education campaigns executed through social media and validated their impact. One such campaign for spreading health care education is The Amyotropic Lateral sclerosis (ALS) Ice Bucket Challenge (Textbox 2).

Textbox 2. **Health care education campaigns through social media.**

1. The ALS Ice Bucket Challenge (2014)

- Overview: Participants dumped buckets of ice water over their heads, shared videos on social media, and nominated others to do the same, all to raise awareness and funds for Amyotrophic Lateral Sclerosis (ALS) research [23].
- Impact:
  - Financial: Raised over US $115 million for the ALS Association in just a few months.
  - Awareness: Dramatically increased global awareness of ALS, with millions participating worldwide.
  - Research Advancement: Funds contributed to significant research advancements, including the discovery of new ALS genes.

### Pakistan Context

In Pakistan, the Federal and provincial governments jointly administer 60% of the health care system, with the private sector contributing 30%. Autonomous bodies support the remaining 10 percent [24]. The diverse structure and the country’s economic challenges pose unique obstacles to effective health care delivery. The country’s health care spending is at 2.95% of its Gross Domestic Product, and the Pakistani government always disregarded the economic survey data and faced criticism from the apex medical organization as it necessitated health allocations in line with global guidelines [25]. To provide quality health care, the Pakistan Medical Association (PMA) expressed the need for 6% of the country’s Gross Domestic Product allocation, as recommended by the World Health Organization [17], which the country is unable to meet due to economic challenges [26].

Due to these financial limitations, there are reported disparities in health awareness, with urban areas generally having better access than rural regions [2]. Thus, communicable diseases, such as waterborne diseases and vector-borne diseases, remain a more significant concern in rural regions [1,3]. Moreover, with limited access to maternal health care and family planning services, Pakistan witnesses high maternal and child mortality rates [2]. Diseases such as malaria, tuberculosis, and hepatitis are prevalent, which exacerbates the situation [24]. Moreover, stigma about sexually transmitted infections results in inadequate prevention and treatment efforts that result in a psychological attack on patients [25]. Poor sanitation and hygiene contribute to outbreaks of diseases like cholera and dysentery in Pakistan and many preventable diseases, such as polio and measles, persist due to low vaccination rates [23].

Amid financial crises, health care awareness and education are known to be effective in reducing the disease burden rate [8]. There are several traditional efforts made by the Government of Pakistan Ministry of Health in the past to improve disease awareness and reduce the patient burden [24]. These include the following:

1. Engaging community health workers in disseminating health information, especially in rural areas. However, the number of community health workers to cover the target population is very limited and requires substantial spending [24].
2. Use of radio and television for health awareness programs, public service announcements, and talk shows but requires financial resources [27].
3. Newspapers, magazines, and pamphlets are also used to spread health information, but considering the low literacy rate and cost of print media, these are not found to be much effective for the target population [27].

However, among the modern techniques, social media has gained much attention from health care communities globally [7,10]. Pakistan has also witnessed the widespread of social media but in academics, e-commerce, entertainment, media, politics, sports, and religious sectors. In Pakistan, 29.5% of the population is on social media, whereas 77.8% has active mobile connections, meaning these users also have access to social media platforms [28].

The health care sector is structured to depend on government approval for any initiative [29]. The government has not devised any legislation to promote the use of social media for awareness [24,27]. This mainly restricts the government’s health departments and health care professionals from using this tool to publicize health messages. Some efforts have been made to digitalize the health care sector using the eHealth concept and implement a few apps to initiate telehealth and telemedicine [30]. Also, mobile messaging and caller voice tunes are found to be significant in health care services awareness [30]. However, the interpretation and usage of social media tools are still scarce [31]. For this reason, it is imperative to investigate the social media usefulness among health care professionals in Pakistan and to identify whether these health care professionals support the use of social media for health awareness and education.

### Theoretical Foundation

A few theories explain the behaviors toward acceptance of any new digitalized tool and address their attitude [32], including the innovation diffusion theory proposed by Rogers in 1962, the Theory of Reasoned Action proposed by Fishbein and Ajzen in 1967, the Self-Efficacy Theory proposed by Bandura in 1977, the Theory of Planned Behavior proposed by Ajzen in 1985, the Social Cognitive Theory proposed by Bandura in 1986, and the Technology Acceptance Model proposed by Davis in 1986 [32]. Among these various behavioral theories, the Innovation Diffusion Theory (IDT) offers a framework for understanding how new ideas, behaviors, or innovations spread within a population [33].

**Figure 1.**
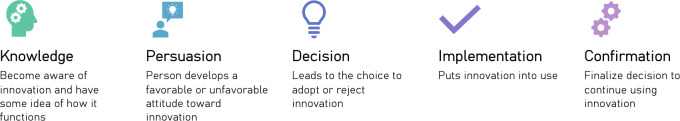
Innovation diffusion process.

In public health, innovation diffusion theory helps explain how new health interventions, practices, or policies are adopted and disseminated within communities [33]. The theory identifies 5 stages individuals and communities pass when adopting an innovation: knowledge, persuasion, decision, implementation, and confirmation [34]. Figure 1 explains these five stages of the innovation-decision process.

**Figure 2.**
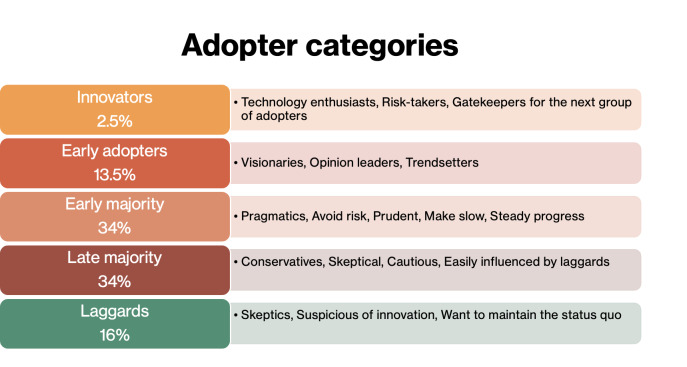
Adopter categories.

To understand social media acceptance for health awareness and education, the theory has categorized individuals within a population into subgroups based on their readiness to adopt innovations. Innovators and early adopters are likely to adopt innovations early, while most of the population follows suit over time. Laggards are the last to adopt [33]. Figure 2 explains these subgroups of individuals.

In health care education and awareness, the diffusion of innovation is used to fast-track the acceptance of crucial public health digitalized interventions that ordinarily intend to influence the behavior of a social system [34]. Thus, this theory provides the foundation for understanding social media usage and its adoption attitude in a particular community.

In Pakistan, the ineffective innovation management approaches and deprived diffusion of innovation strategies are hurdles to realizing the importance of social media and its usage for health care education [24,25]. The deprived diffusion of innovation is a significant factor in the inability to attain the desired output. Even for digitalized hospital tools other than social media, the country needs more innovative and user-friendly equipment in the hospital sector [25]. The notion of consistent health care challenges and inadequate health resources in Pakistan is also evident from the recent Joint External Evaluation report [24].

For this reason, it is imperative to investigate the impact of social media’s effective use on health awareness and health care education in Pakistan using IDT. This may result in highlighting and understanding the effectiveness of social media tools in reducing the disease burden in the country.

**Figure 3.**
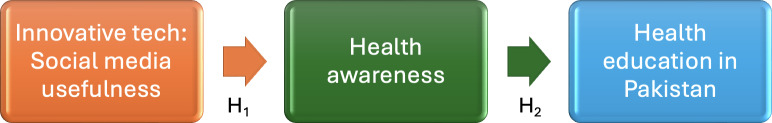
Conceptual framework of the study.

Figure 3 explains the theoretical framework of the current study.

Hence, the current study hypotheses are:

1. Hypothesis 1 (H1): social media usefulness positively and significantly impacts health awareness in Pakistan.
2. Hypothesis 2 (H2): health awareness has a significant positive impact on health care education in Pakistan.

## Methods

### Instrument Development

A quantitative technique was applied to test the study hypothesis and validate the proposed model using the structural equation modeling (SEM) technique [35]. SEM is deemed the most suitable due to its ability to simultaneously test complex relationships among multiple variables and validate the proposed conceptual model. Data analysis was carried out using AMOS 26.0 software (IBM Corp) [36]. Since the study aims to identify social media’s impact on health awareness leading to health care education and uses the IDT as a theoretical foundation, scales from previous literature have been adopted. The main advantage of adopting these scales is that they have already been validated in the health industry’s acceptance behavior of new technological tools. For instance, the measurement scale for Innovative Technology (social media) usefulness was adopted from a previous study [37], and measurement scales for health awareness have been adopted from another study [38]. Aligning with the psychometric literature, 4-item scales for each construct based on their higher loadings have been adopted [37]. The modified 8-item scale for measuring health care education was adopted based on the questionnaire of Ho et al [39]. For the measurement of each item, a 7-point Likert scale ranging from 1 (strongly disagree) to 7 (strongly agree) was used [38].

### Data Collection

A questionnaire was designed based on the variables of the present study, utilizing the aforementioned scales, which are provided in Multimedia Appendix 1. A personally administered questionnaire was used for data collection [32]. For data collection, the current research to eliminate targeted health care professionals and paramedic staff of 2 private hospitals in Lahore, Pakistan. The questionnaire was distributed to more than 450 health care professionals and paramedic staff using convenience sampling and requested their valuable feedback. However, only 389 useful responses were received.

### Ethical Considerations

As a matter of ethical concerns, prior permission from the hospital management was obtained, and the purpose of the study was explained to all respondents through “informed consent.” It was assured that respondents’ privacy would be ensured through confidentiality. Ethical approval was not required for this study as it did not involve identifiable personal data, or interventions falling under the scope of institutional or national ethics review requirements. In accordance with the Declaration of Helsinki (World Medical Association, 2013) research ethics policy, studies that do not involve sensitive data are exempt from formal ethics board approval.

## Results

### Primary Data Screening

Initial screening was carried out among 389 useful responses to eliminate unusual responses, mainly incomplete questionnaires and respondents who marked similar scores on all items. Furthermore, questionnaires with more than 4 missing values were also excluded. The remaining missing values were replaced with the series mean/average for the rest of the responses. Therefore, the final useful sample was reduced to 340.

Table 1 provides the demographics of the study respondents. Among 340 respondents, 58% (197/340) were male, and the rest were female (143/340, 42%). A total of 91% (309/340) of the respondents were between 25 and 35 years of age, which may lead to the fact that most health care professionals and paramedic staff in private hospitals in Lahore are young. However, we may be unable to neglect the 6% of the population under 25 years old and more eager to start their career in the health care sector. The remaining 3% were more than 35 years of age and had extensive health industry work experience.

**Table 1.** Sociodemographic characteristics of study respondents (N=340).

Characteristics		Values, n (%)	
Sex			
Male		197 (58)	
Female		143 (42)	
Age (years)			
<25		21 (6)	
25-35		309 (91)	
>35		10 (3)	
Social media experience (years)			
<1		34 (10)	
2-5		207 (61)	
>5		99 (29)	
Monthly income^a^			
Less than PKR^b^ 40,000 (US $142.98)		101 (30)	
PKR 40,000-80,000 (US $142.98-US $285.97)		197 (58)	
More than 80,000 (US $285.97)		42 (12)	
Education			
Less than secondary school		63 (18)	
Secondary school to graduation		259 (76)	
Medical (MBBS^c^/BDS)^d^ or Masters degree		18 (6)	
Health services experience (years)			
<3		21 (6)	
3-6		225 (66)	
>6		94 (28)	

a A currency exchange rate of US $1=280 PKR is applicable.

b PKR: Pakistani rupees.

c MBBS: Bachelor of Medicine, Bachelor of Surgery.

d BDS: Bachelor of Dental Surgery.

### Data Analysis

An exploratory factor analysis was initially performed on the 16 measurement items to confirm the underlying relationships [36]. For sample adequacy, the KMO value (0.850) was greater than the recommended value (0.60), and the significance value of *P*0.001 confirmed the Bartlett test of sphericity, as shown in Table S1 in Multimedia Appendix 2. All those factors were retained for data analysis, with factor loading greater than 0.50 and Eigenvalue greater than 1.0. Hence, this technique provided 3 factors that explained 72.2% of the variance after varimax rotation. Furthermore, scale reliability coefficients were greater than the acceptable value (0.70) [40], and Shapiro-Wilk tests were conducted to check the data normality [40], as shown in Table S2 in Multimedia Appendix 2 . The results of both tests were significant, meaning there was no normality issue in the data.

For further data analysis, a two-stage structural equation modeling (SEM) technique was used [36-38]. This approach allows researchers to evaluate the measurement and structural models separately using two dissimilar subsamples. For this reason, a sample of 340 participants was divided into two parts: a sample of 170 individuals was used for the measurement model, and a similar sample size was used for the structural model assessment to attain impartial results.

### Reliability and Validity Measures

According to Hair et al [35], finding the reliability of each item and construct in the research study is essential. Therefore, squared multiple correlation (SMC) was used to find the reliability of each reliability item of each measurement item. SMC represents “the amount of variance explained by an individual indicator/construct of its respective factor and measured by the square of its (indicator’s) standardized factor loading” [40].

Hair [35] indicated that the cutoff value of SMC is 0.30. Hence, it is evident from Table 2 that all the SMC values are greater than 0.30. For measuring the reliability of each variable, the Cronbach alpha value was used. As suggested in the literature, the cutoff value of Cronbach alpha is 0.70 [36,37]; it is evident from Table 2 that all variables’ Cronbach alpha values are more than 0.70. Furthermore, Hair et al [35] also highlighted that for measuring the convergent validity, there are three common approaches: (1) standardized factor loading (0.5 or greater), (2) average variance explained (0.5 or higher), and (3) composite reliability (0.7 or above).

**Table 2.** Measurement of reliability and validity.

Constructs and item		Values, mean (SD)		Standardized factor loading	*S*quared multiple correlation	Composite reliability	Cronbach alpha	Average variance explained
Social media usefulness (SMU)								
SMU1		5.19 (1.65)		0.781	0.607	0.788	0.774	0.589
SMU2		5.33 (1.71)		0.780	0.687			
SMU3		5.01 (1.12)		0.777	0.671			
SMU4		5.05 (1.32)		0.787	0.646			
Health awareness (HA)								
HA1		5.45 (1.56)		0.859	0.677	0.769	0.773	0.592
HA2		5.98 (1.45)		0.874	0.681			
HA3		5.39 (1.43)		0.881	0.675			
HA4		5.51 (1.39)		0.867	0.654			
Health care education (HED)								
HED1		5.23 (1.77)		0.719	0.651	0.783	0.789	0.678
HED2		5.22 (1.74)		0.775	0.629			
HED3		5.60 (1.64)		0.721	0.622			
HED4		5.41 (1.49)		0.739	0.637			
HED5		5.22 (1.15)		0.787	0.663			
HED6		5.11 (1.23)		0.772	0.654			
HED7		5.09 (1.43)		0.759	0.691			
HED8		5.32 (1.59)		0.768	0.609			

Hence, each standardized factor loading was statistically significant (*P*<.001), and values ranged from 0.719 to 0.881, thus validating adequate convergent validity as shown in Table 2. In addition, the average value explained (AVE) values and construct reliability were also more than their cut-off level of 0.5 and 0.7, respectively, and were statistically significant. Therefore, these measures confirm sufficient convergent validity.

A comparison of shared variance between factors with the average variance explained by individual factors was ensured for measuring the discriminant validity. The diagonal value should be greater than the non-diagonal value to confirm adequate discriminant validity [40]. Hence, results given in Table S3 in Multimedia Appendix 2indicates the correlation matrix of constructs, where non-diagonal elements are correlated among constructs and diagonal elements are the square root of average variance explained (AVE) by that construct and clearly explain that all three constructs differ.

### Measurement Model

Using statistical software AMOS 26.0, the measurement model’s confirmatory factor analysis (CFA) was performed [40,41]. This provided a passable model fit for the primary measurement model (CMIN=1220.23; CMIN/DF=1.98; GFI=0.72; AGFI=0.80; NFI=0.81; CFI=0.826; RMR=0.089; RMSEA=0.082); however, the number of indicators per item was large; for instance, the number of indicators for health care education were 8. Consequently, 2 items were deleted for further refinement to obtain an adequate model fit through the CFA of the measurement model. This refinement was conducted by deleting items one by one, based on their standardized residual; that is, that item was first deleted, which had a larger error variance than their measurement items. Each item was carefully reviewed before deleting it to ensure that, from a theoretical viewpoint, its error variance also seemed rational. The refinement and assessment process for every construct was first evident by Churchill [41]. Churchill defined this process as; “Though this application may be satisfactory during the early stages of research on a construct, the use of factor analysis in a confirmatory fashion would seem better at later stages.” Furthermore, Gerbing and Anderson’s [42] study also provided support to Churchill’s argument and stated that; “to demonstrate that an explicit evaluation of Unidimensionality is accomplished with a confirmatory factor analysis of the individual measures as specified by a multiple-indicator measurement model. Coefficient alpha is important in assessing reliability but does not assess dimensionality. Although item-total correlations and exploratory factor analysis can provide useful preliminary analyses, particularly in the absence of a sufficiently detailed theory, they do not directly assess unidimensionality. The reason is that a confirmatory factor analysis assesses the internal consistency and external consistency criteria of unidimensionality implied by the multiple-indicator measurement model.

Hence, this refinement process provided an adequate model fit (CMIN=357.62; the ratio of *χ*^2^ to degree of freedom value (1.80) is remarkably less than its recommended value (5.0). CMIN/DF=1.80; GFI=0.90; AGFI=0.89; NFI=0:915; CFI=0:93; RMR=0:075; RMSEA=0:055) as shown in Table 3.

**Table 3.** Structural equation modeling fit indices for the confirmatory factor analysis model.

Fit indices	Cut-off criteria	Results obtained
Absolute fit indices		
Chi-square (*df*)	N/A^a^	357.62 (170)
*χ*^2^/df (CMIN/DF)	<5.00	1.800
Root Mean Square Error of Approximation	<0.06	0.055
Goodness of Fit Index	>0.85	0.900
Adjusted Goodness of Fit Index	>0.85	0.890
Incremental fit indices		
Buntler-Bonett Normed Fit Index	>0.90	0.915
Comparative Fit Index	>0.93	0.930
Tucker Lewis Index	>0.90	0.941
Incremental Fit Index	>0.90	0.932
Parsimonious fit indices		
Parsimony Goodness-Fit Index	>0.50	0.798
Parsimony Normed Fit Index	>0.50	0.848

a N/A: not applicable.

### Structural Model

The research hypotheses were tested through structural model estimation [40]. Therefore, a second subsample (n=170) was used and provided the adequate structural model fit (CMIN=488.6; CMIN/DF=1.85; GFI=0.861; AGFI=0.893; NFI=0.987; CFI=0.945; RMR=0:079; RMSEA=0.053) as shown in Table 4.

**Table 4.** Hypothesized structural model fit indices.

Fit index	Cut-off criteria	Results obtained
Absolute fit indices		
Chi-square (*df*)	N/A^a^	488.6 (170)
*χ*^2^/df (CMIN/DF)	<5.00	1.850
Root Mean Square Error of Approximation	<0.06	0.053
Goodness of Fit Index	>0.85	0.861
Adjusted Goodness of Fit Index	>0.85	0.893
Incremental fit indices		
Buntler-Bonett Normed Fit Index	>0.90	0.987
Comparative Fit Index	>0.93	0.945
Tucker Lewis Index	>0.90	0.976
Incremental Fit Index	>0.90	0.943
Parsimonious fit indices		
Parsimony Goodness-Fit Index	>0.50	0.680
Parsimony Normed Fit Index	>0.50	0.609

a N/A: not applicable.

Moreover, the significance of the hypothesis is shown in Table 5. Testing of H1 revealed that social media usefulness has a significant effect on health awareness (H1: *β*=.669, *P*<.001), supporting the IDT proposed by Rogers. Testing of H2 also supplied similar significant outcomes to the current research model (H2: *β*=.557, *P*<.001), confirming the findins of prior work.

**Table 5.** Hypothesis testing results.

S.no.	Impact of	Impact on	Hypothesis	Path coefficient	*P* value	Outcome
1	SMU^a^	HA^b^	H1^c^	0.669^d^	.001	Significant impact
2	HA	HED^e^	H2^f^	0.557^d^	.001	Significant impact

a SMU: social media usefulness.

b HA: health awareness.

c H1: hypothesis 1.

d *P*<.001.

e HED: health care education.

f H2: hypothesis 2.

## Discussion

### Principal Findings

This study examined the impact of social media on health awareness and its role in health care education, using IDT as the theoretical framework in Pakistan. A conceptual model was developed to assess the mediating effect of health awareness on the relationship between social media usage and health care education. The hypothesis testing results demonstrated significant relationships, with social media positively influencing health awareness (H_1_: path coefficient=0.669, *P*<.001) and health awareness positively impacting health care education (H_2_: path coefficient=0.557, *P*<.001). These findings highlight the potential of social media as an effective tool for promoting health care education when strategically leveraged by health care stakeholders.

The study also revealed that younger and more educated health staff are more inclined to use social media, while older groups, though recognizing its usefulness, engage less actively. This demographic insight underlines the need for tailored engagement strategies to maximize participation. Limited research in developing countries, including Pakistan, has tested the application of IDT to health care professionals’ adoption of social media for health care education. The standardized direct effects from hypothesis testing affirm the significant relationship between social media usefulness, health awareness, and health care education in the Pakistani context.

Given that developing countries are often classified as late adopters of technology, according to IDT, the findings emphasize the importance of region-specific social media content on platforms, such as Facebook, WhatsApp, or locally developed alternatives, for health care interventions. IDT further suggests that simplifying innovations can facilitate broader adoption, making it easier for health care professionals and the general population to engage with digital health initiatives. These insights highlight the need for cost-effective, user-centric solutions to enhance healthcare outcomes in Pakistan.

The government of Pakistan can also play a significant role in spreading health awareness. They can use social media to run campaigns on maternal health, child nutrition, and disease prevention. The health care professionals in private hospitals also indicated that live sessions and webinars on platforms like Facebook Live and Instagram Live to educate the patients and hospital staff on various health issues and answer their real-time questions have been very useful.

Using social media for health care education in Pakistan could be a game-changer, surpassing the expectations of spreading awareness regarding health needs in a particular community. This potential is not just theoretical; it has been proven in developed countries, such as the US, Jordan, the United Kingdom, and Europe, where social media was used during the COVID-19 pandemic to combat the pandemic and other notable diseases [15]. Moreover, social media platforms have been used to promote telemedicine services, enabling health care professionals to connect with patients remotely for consultations, follow-ups, and monitoring of chronic conditions [16,30].

Overall, this study validates the use of social media to improve public health care education through public awareness. Therefore, the study concludes that health care professionals should use social media tools to inform the wider public and address healthcare issues in Pakistan.

### Conclusion

The findings of this study underscore the significant role that social media plays in enhancing health care awareness and education among various populations in developing countries like Pakistan. With their wide reach and interactive features, social media platforms have proven to be effective tools for disseminating health-related information and engaging individuals in meaningful health conversations. Social media breaks down geographical barriers, allowing health information to reach a global audience instantly. This broadens the scope of health care awareness campaigns and makes information accessible to a diverse demographic, including those in remote or underserved areas. The interactive nature of social media facilitates active engagement between health care providers and the public. This interaction fosters a sense of community, encourages the sharing of personal health experiences, and allows for immediate feedback and clarification of health information. Also, social media platforms enable the dissemination of personalized health information tailored to specific audiences’ needs. Moreover, the real-time nature of these platforms ensures that information is up to date, which is crucial during health emergencies or outbreaks.

Health care education through social media can help understand how to prevent common diseases such as malaria, tuberculosis, hepatitis, and polio through vaccination, sanitation, and hygiene in Pakistan. Awareness campaigns can encourage healthier lifestyles, reducing the incidence of chronic diseases such as diabetes, hypertension, and heart disease. Educating women about prenatal and postnatal care, safe delivery practices, and child nutrition can significantly lower maternal and infant mortality rates. Awareness through social media about proper nutrition, immunization, and hygiene practices helps improve children’s health and development in the rural outskirts of Pakistan. Also, awareness about communicable diseases and their transmission can help in preventing outbreaks of diseases like HIV/AIDS, tuberculosis, and waterborne illnesses.

Governmental policies provide a framework for regulating the content shared on social media, ensuring that the information disseminated is accurate, reliable, and in line with public health guidelines. Information shared by government-endorsed social media accounts is more likely to be trusted by the public. Official policies lend credibility and legitimacy to health messages, increasing the likelihood of public acceptance and compliance. All active organizations (govt and private) working in the health industry of Pakistan seek prior permission or ethical clearance to initiate any new work. Without government support, the organizations lack the confidence to spread and advertise any health-related content due to fear that they may be subject to local resistance.

Despite its benefits, the use of social media in health care awareness also presents challenges, such as the spread of misinformation and the need for privacy and data protection. Health care organizations must implement strategies to verify information and ensure the credibility of the content shared. Hence, the study suggests that Pakistan’s health care organizations should continue leveraging social media to enhance health communication strategies and education. Ongoing research is also needed to explore new ways to maximize social media’s benefits while mitigating its risks.

In conclusion, social media is a powerful tool for health care awareness, offering unprecedented opportunities to reach and engage with a wide audience. By harnessing its potential in developing economies, health care organizations can improve public health literacy, promote healthy behaviors, and ultimately contribute to better health outcomes. Collaborative efforts between health care professionals, social media platforms, and policymakers will be crucial in leveraging social media’s full potential for public health advancement.

### Future Research

Future research should move beyond compliance with existing social media policies to explore strategies and variables that demonstrate tangible improvements in the health care sector. This could include investigating how social media can influence enhanced patient outcomes through better communication and engagement, broaden health awareness campaigns by targeting diverse and underserved populations, and improve access to health care services through innovative digital solutions. Additionally, longitudinal studies examining the long-term impact of social media interventions on public health indicators would provide valuable insights. Identifying and analyzing these variables will contribute to a more comprehensive understanding of social media’s transformative potential in health care beyond mere policy adherence.

## Supplementary material

10.2196/65745 Multimedia Appendix 1 Questionnaire.

10.2196/65745 Multimedia Appendix 2 Additional tables.

## Abbreviations

AGFI: Adjusted Goodness of Fit Index
AVE: average value explained
CFA: confirmatory factor analysis
CFI: Comparative Fit Index
CMIN: chi-square minimum
DF: degrees of freedom
GFI: Goodness of Fit
H1: hypothesis 1
H2: hypothesis 2
IDT: Innovation Diffusion Theory
NFI: Buntler-Bonett Normed Fit Index
PMA: Pakistan Medical Association
RMR: Root Mean Square Residual
RMSEA: Root Mean Square Error of Approximation
SEM: structural equation modeling
SMC: squared multiple correlation
