# Supplementary material for: Using Social Media Platforms to Raise Health Awareness and Increase Health Education in Pakistan: Structural Equation Modeling Analysis and Questionnaire Study
Source: JMIR Hum Factors. 2025 Apr 7;12:e65745. doi: 10.2196/65745 (PMC11996147; doi:10.2196/65745)
Supplement: Multimedia Appendix 1 [file humanfactors-v12-e65745-s001.docx]

**Questionnaire**

Socio-demographic factors:

1. Name: ______________________ (You can leave this blank if you don’t want to provide your name)

2. Gender:

□ Male

□ Female

3. Age:

□ Less than 25 years

□ 25 – 35 years

□ More than 35 years

4. Social Media Experiences:

□ Less than 01 year

□ 02 to 05 years

□ More than 35 years

5. Health Services Experience:

□ Less than 03 years

□ 03 to 06 years

□ More than 06 years

6. Education:

□ Less than Secondary School

□ Secondary School to Graduation

□ Medical Degree (MBBS/BDS) or Masters

7. Monthly Income:

□ Less than Rs. 40,000

□ Rs. 40,000 to Rs. 80,000

□ More than Rs. 80,000

In the below Likert scale, SD (Strongly Disagree = 1), D (Disagree = 2), SD (Somewhat Disagree = 3), N (Neutral = 4), SA (Somewhat Agree = 5), A (Agree = 6), (Strongly Agree =7)

(Please check the box to record your answer against each question statement)

| **“S. #** | **Question Statements** | **LIKERT SCALE** | | | | | | |
| --- | --- | --- | --- | --- | --- | --- | --- | --- |
|  |  | **SD (1)** | **D (2)** | **SD (3)** | **N (4)** | **SA (5)** | **A (6)** | **SA (7)** |
| 8. | Social Media tools (Facebook, Tiktok, Twitter, Instagram, YouTube etc) are easy to learn and navigate. |  |  |  |  |  |  |  |
| 9. | Social Media tools (Facebook, Tiktok, Twitter, Instagram, YouTube etc) generally helps me to find the relevant contents in a convenient way. |  |  |  |  |  |  |  |
| 10. | Social Media tools (Facebook, Tiktok, Twitter, Instagram, YouTube etc) are productive for easy access of larger information. |  |  |  |  |  |  |  |
| 11. | Social Media tools (Facebook, Tiktok, Twitter, Instagram, YouTube etc) are flexible and offers support in socializing with other people. |  |  |  |  |  |  |  |
| 12. | I understand the social media language and I use it to enjoy interesting contents. |  |  |  |  |  |  |  |
| 13. | The social media features are easy to navigate and most of their tools are user-friendly. |  |  |  |  |  |  |  |
| 14. | I have complete awareness of social media and what are the pros and cons associated with its usage. |  |  |  |  |  |  |  |
| 15. | I am aware about the procedure how to create my personal account and how to obtain the login details on social media. |  |  |  |  |  |  |  |
| 16. | Social medial tools can be used to spread the awareness and education in country. |  |  |  |  |  |  |  |
| 17. | I get the chance to learn many new developments in my health filed through social media. |  |  |  |  |  |  |  |
| 18. | I can see contents and my profession related videos whenever I want through social media. |  |  |  |  |  |  |  |
| 19. | Health professionals can use this social media to spread education among people |  |  |  |  |  |  |  |
| 20. | I have seen a few videos and contents from health professionals spreading the awarness through social media. |  |  |  |  |  |  |  |
| 21. | People of my field enjoy using social media for entertainment as well as for education purposes. |  |  |  |  |  |  |  |
| 22. | Social Media tools (Facebook, Tiktok, Twitter, Instagram, YouTube etc) can be used to spread the awareness and education for various health diseases in the country. |  |  |  |  |  |  |  |
| 23. | Social Media tools (Facebook, Tiktok, Twitter, Instagram, YouTube etc) can be effective to spread awarness and education to reduce disease burden in the country. |  |  |  |  |  |  |  |
