# Supplementary material for: Using Social Media Platforms to Raise Health Awareness and Increase Health Education in Pakistan: Structural Equation Modeling Analysis and Questionnaire Study
Source: JMIR Hum Factors. 2025 Apr 7;12:e65745. doi: 10.2196/65745 (PMC11996147; doi:10.2196/65745)
Supplement: Multimedia Appendix 2 [file humanfactors-v12-e65745-s002.docx]

Table S1. Keyser-Meyer-Olkin (KMO) and Bartlett’s test.

| Kaiser-Meyer-Olkin measure of sampling adequacy | | | | | | 0.850 |
| --- | --- | --- | --- | --- | --- | --- |
|  |  |  |  |  |  |  |
| Bartlett’s test of sphericity | | |  | Approx. chi-square | | 19674.23 |
|  |  |  |  | Df |  | 340 |
|  |  |  |  | Significance |  | 0.001 |

Table S2. Latent constructs Kolmogorov-Smirnov and Shapiro-Wilk test.

|  | Kolmogorov-Smirnov^a^ | | | Shapiro-Wilk | | |
| --- | --- | --- | --- | --- | --- | --- |
|  | Statistic | Df | Significance | Statistic | Df | Significance |
| Social media usefulness | 0.098 | 340 | 0.000 | 0.901 | 340 | 0.000 |
| Health awareness | 0.109 | 340 | 0.000 | 0.923 | 340 | 0.000 |
| Health care education | 0.126 | 340 | 0.000 | 0.966 | 340 | 0.000 |

^a^Lilliefors Significance Correction.

Table S3. Measurement of discriminant validity.

| Construct | SMU | HA | HED |
| --- | --- | --- | --- |
| SMU | 0.791 |  |  |
| HA | 0.663 | 0.701 |  |
| HED | 0.423 | 0.523 | 0.711 |
